# 

**Published:** 1846-12

**Authors:** 


### CONTENTS

OF NO. VI.

### ORIGINAL COMMUNICATIONS.

### ESSAYS AND CASES.

ART. I.—Observations on the Medical Topography, Climate, and Endemic Influences of South Alabama. By JAMES C. HARRIS, M.D., of Wetumpka, Ala. - 461

ART. II.—A Case of Puerperal Mania from Suppression of the Lochia; and one of Mania a Potu complicated with Pleurisy. By JAMES M. LARKINS, M.D., of Charlotte, Tenn. - - - - - - - - 474

ART. III.—A Case of Tubercles of the Brain. By WM. C. SNEED, M.D., of Frankfort, Ky. - - - 476

ART. IV.—Notes on Medical Matters in Paris. By DAVID W. YANDELL, M.D., of Louisville, Ky. - - 479

### REVIEWS.

ART. V.—The Writings of Hippocrates and Galen. Epitomised from the Original Latin Translations. By JOHN REDMAN COXE, M.D., Member of the Batavian Society of Sciences at Haarlem; &c., &c. - 485

ART. VI.—Chemistry of the Four seasons: Spring, Summer Autumn, Winter; an Essay principally concerning natural phenomena admitting of interpretation by Chemical Science, and illustrating passages of Scripture. By THOMAS GRIFFITHS, Professor of Chemistry in the Medical College of St. Bartholomew's Hospital; author of 'Recreations in Chemistry,' and 'Chemistry of the Four Elements.' - - - 516

ART. VII.—An Anatomical Description of the Diseases of the Organs of Circulation and Respiration. By CHARLES EWALD HASSE, M. D., Professor of Pathology and Clinical Medicine at the University of Zurich, etc., etc. Translated and Edited by W. E. SWAINE, M.D., Physician Extraordinary to H. R. H. the Duchess of Kent. - - - - - - - 519

SELECTIONS FROM AMERICAN AND FOREIGN JOURNALS.

On the Use of Muriate of Barytes in Scrofulous Affections. 521
Cod-Liver Oil in Strumous Affections. - - - - - 522
Bromine and its Prepararions. - - - - - - 523
Stramonium Cigars. - - - - - - - - 525
Treatment of Warts. - - - - - - - - 525
Opium in Hemorrhage, - - - - - - - - 525
On the Composition of Air at different heights in close Apartments. - - - - - - - - - - 526
On the Blood in Bodies Killed by Strangling. - - - 527
Sulphate of Iron for Excessive Perspiration. - - - 528
Upas Tree. - - - - - - - - - - 528
Statistics of Medical Schools. - - - - - - 529
Address to the Medical Profession. . - - - - 530
Aneurism Spontaneously Cured. - - - - - - 533
Report upon the Hemostatic Virtues of the Brocchieri Water and Ergotine. - - - - - - - - - 534
Compound Fracture of the Cranium—Loss of a portion of the Brain—Recovery. - - - - - - - - 535
Treatment of Cataract. - - - - - - - - 538
Treatment of Nervous Constipation. - - - - 538
Poisoning by Arsenic. - - - - - - - - 538
Poisoning by Lead-Shot left in a Bottle. - - - 540
Cholera in Holland. - - - - - - - - 540
Two ounces of Nitrate of Potash taken at once. - - 541
Digestion in 1846. - - - - - - - - - 541
Cholera in Persia. - - - - - - - - - 542
Delirium Tremens in an Infant. - - - - - 542
Cholera at Kurrachee. - - - - - - - - 543
Chronic Articular Inflammations. - - - - - - 543
Asiatic Cholera. - - - - - - - - - 544
Anecdote of the Pay of Physicians. - - - - - 544

ORIGINAL INTELLIGENCE.

University of Louisville. - - - - - - - - 546
Progress of Dental Surgery. - - - - - - - 547

